# The Seasonality of Tuberculosis, Sunlight, Vitamin D, and Household Crowding

**DOI:** 10.1093/infdis/jiu121

**Published:** 2014-03-04

**Authors:** Tom Wingfield, Samuel G. Schumacher, Gurjinder Sandhu, Marco A. Tovar, Karine Zevallos, Matthew R. Baldwin, Rosario Montoya, Eric S. Ramos, Chulanee Jongkaewwattana, James J. Lewis, Robert H. Gilman, Jon S. Friedland, Carlton A. Evans

**Affiliations:** 1 IFHAD: Innovation For Health and Development, United Kingdom; 2 Asociación Benefica Prisma; 3 Laboratorio de Investigación y Desarrollo, Universidad Peruana Cayetano Heredia, Perú; 4 Infectious Diseases and Immunity, Imperial College London; 5 Wellcome Trust Imperial College Centre for Global Health Research, London; 6 The Monsall Infectious Diseases Unit, North Manchester General Hospital, Manchester; 7 Medical Research Council Tropical Epidemiology Group, London School of Hygiene and Tropical Medicine, London, United Kingdom; 8 School of Public Health, Johns Hopkins Bloomberg School of Hygiene and Public Health, Baltimore, Maryland

**Keywords:** crowding, household, seasonality, sunlight, tuberculosis, vitamin D

## Abstract

***Background.*** Unlike other respiratory infections, tuberculosis diagnoses increase in summer. We performed an ecological analysis of this paradoxical seasonality in a Peruvian shantytown over 4 years.

***Methods.*** Tuberculosis symptom-onset and diagnosis dates were recorded for 852 patients. Their tuberculosis-exposed cohabitants were tested for tuberculosis infection with the tuberculin skin test (n = 1389) and QuantiFERON assay (n = 576) and vitamin D concentrations (n = 195) quantified from randomly selected cohabitants. Crowding was calculated for all tuberculosis-affected households and daily sunlight records obtained.

***Results.*** Fifty-seven percent of vitamin D measurements revealed deficiency (<50 nmol/L). Risk of deficiency was increased 2.0-fold by female sex (*P* < .001) and 1.4-fold by winter (*P* < .05). During the weeks following peak crowding and trough sunlight, there was a midwinter peak in vitamin D deficiency (*P* < .02). Peak vitamin D deficiency was followed 6 weeks later by a late-winter peak in tuberculin skin test positivity and 12 weeks after that by an early-summer peak in QuantiFERON positivity (both *P* < .04). Twelve weeks after peak QuantiFERON positivity, there was a midsummer peak in tuberculosis symptom onset (*P* < .05) followed after 3 weeks by a late-summer peak in tuberculosis diagnoses (*P* < .001).

***Conclusions.*** The intervals from midwinter peak crowding and trough sunlight to sequential peaks in vitamin D deficiency, tuberculosis infection, symptom onset, and diagnosis may explain the enigmatic late-summer peak in tuberculosis.

Although the incidence of certain infectious diseases is seasonal [1], the seasonality of tuberculosis is incompletely understood.

Tuberculosis infects approximately a third of the world's population, causing symptomatic disease in 8.7 million people annually [2]. Prior to antibiotics, spring peaks were noted in tuberculosis illness [3]. Later studies in the antibiotic era from Cameroon [4], India [5], Britain [6], Kuwait [7], Spain [8], America [9], Japan [10], and South Africa [11, 12] also revealed tuberculosis seasonality. Contrary to recognized patterns of acute respiratory illnesses [13], most studies report a nadir of new tuberculosis cases during winter and a peak in spring and summer. This seasonal variation is presumed to relate more to recent transmission than tuberculosis reactivation [12, 14]. However, interpretation of seasonality studies is complicated by heterogeneity in definition of season and seasonal variables studied (ie, temperature, rainfall) [4, 7, 11].

Vitamin D is an important determinant of adaptive and innate immunity. The principal active metabolite of vitamin D, 25-hydroxy-vitamin D (hereafter referred to as “vitamin D”), has immunosuppressive effects on T-helper and dendritic cells but, conversely, an immunostimulatory effect on monocytes and macrophages [15–17]. Low vitamin D concentrations contribute to an increased risk of tuberculosis contacts' tuberculin skin test (TST) converting to positive [18] and a higher likelihood of active tuberculosis disease in people with specific vitamin D receptor polymorphisms [19]. Apart from diet, humans derive a proportion of their vitamin D through synthesis from 7-dehydrocholesterol on exposure of skin to sunlight [20]. Historically, both ultraviolet light and vitamin D supplementation were used in the treatment of pulmonary and cutaneous tuberculosis [21]. More recently, vitamin D supplementation during tuberculosis treatment was investigated but did not improve treatment outcome [22, 23]. This tuberculosis and vitamin D interaction has stimulated interest in seasonal variation in vitamin D concentrations as a potential risk factor for tuberculosis susceptibility [11, 14, 22–25].

Poverty and social determinants are associated with tuberculosis infection and disease [26–28]. In diverse settings, household crowding (hereafter referred to as “crowding”) is associated with poverty, tuberculosis infection, and tuberculosis disease in household contacts [29–32].

Understanding tuberculosis seasonality and its potential associations with both endogenous and exogenous factors, including vitamin D and crowding, may inform the health effects of climate change and influence tuberculosis prevention through interventions to reduce crowding and vitamin D deficiency. We therefore studied the seasonal relationship between putative tuberculosis risk factors (crowding, hours without sunlight, and vitamin D concentrations), tuberculosis infection (measured by TST and interferon-γ release assays [IGRAs]), and subsequent tuberculosis illness (symptom onset and disease) within an impoverished community in northern Lima, Peru.

## METHODS

### Design Overview

This was an ecological analysis of seasonality conducted during a cohort study investigating risk factors for incident tuberculosis amongst household contacts of tuberculosis patients [27]. An ongoing nested trial of micronutrient supplementation is assessing whether micronutrient supplementation prevents tuberculosis, but all data reported here involved participants who declared they had not taken micronutrient supplements.

For the current research, the tuberculosis patients had their date of symptom onset and diagnosis recorded and their tuberculosis-exposed cohabitants were tested for tuberculosis infection and vitamin D deficiency while sunlight levels were recorded.

### Setting and Climate Data

The study took place over 4 years from 1 January 2003 until 31 December 2006 in Ventanilla, a periurban shantytown with high rates of tuberculosis disease (162/100 000/year) and poverty, but low rates (<2%) of HIV-tuberculosis coinfection [27].

Hours without direct sunlight were studied because we hypothesized that these would be associated with vitamin D deficiency. The Peruvian Ministry for Environment (SENAMHI) provided data defining the presence or absence of direct sunlight in the area over the 32 640 consecutive hours of the study period. Direct sunlight was present during daylight hours when cloud cover was minimal or absent. Conversely, direct sunlight was absent during hours of darkness or when cloud cover prevented the measuring apparatus from directly receiving sunlight. Lima has 2 main seasons: we defined winter as the 6 consecutive months with fewest hours of direct sunlight. Summer was defined as the rest of the year.

### Participants

**Figure 1.**
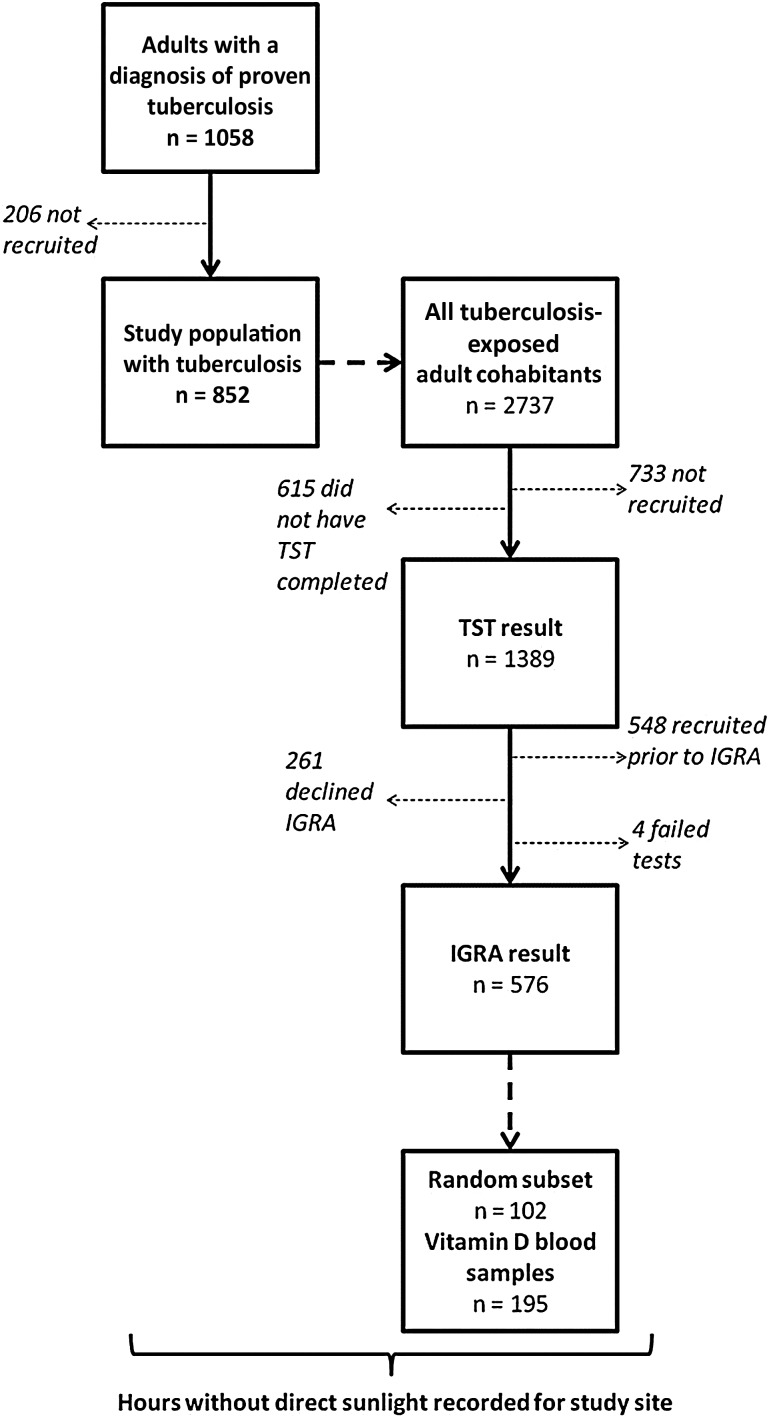
Study design. Abbreviations: IGRA, interferon-γ release assay; TST, tuberculin skin test.

Inclusion criteria were adult tuberculosis patients with laboratory-proven (sputum smear or culture positive) pulmonary tuberculosis and their adult tuberculosis-exposed household cohabitants. Adults in this setting were defined as aged 16 years or older. Tuberculosis-exposed cohabitants were individuals who reported being in the same house as these tuberculosis patients for over 2 hours per day at least 3 times per week. Exclusion criteria were declining/inability to give informed written consent (Figure 1).

The national tuberculosis program registered 1058 patients with pulmonary tuberculosis during the study period. We located 99%, and 93% (n = 852) of those who met our inclusion criteria (n = 912) consented to participate. We concurrently aimed to recruit all tuberculosis-exposed cohabitants of these patients, and 73% (n = 2,004) of those who met our inclusion criteria (n = 2737) consented to participate. Thus, 3589 members of tuberculosis-affected households were recruited. The internationally accredited ethics committee of the Universidad Peruana Cayetano Heredia approved the project.

### Procedures

At enrolment, a questionnaire was completed with all participants to record baseline data (Table 1). For tuberculosis patients, this questionnaire included information defining the date of symptom onset (see Table 2) and the date of diagnosis.

**Table 1.** Study Population Baseline Data

	Tuberculosis-Exposed Cohabitants		Tuberculosis Patients
	All	Randomly Selected Individuals for Plasma Vitamin D Measurement	All
Number of participants	1389	102	852
Number of blood vitamin D analyses	N/A	195	N/A
Demographics			
Sex, % males (95% CI)	37 (34–39)	31 (25–38)	60 (56–63)
Age, mean years (95% CI)	34 (33–34)	34 (32–36)	31 (30–32)
Socioeconomic factors			
Any postprimary education, % (95% CI)	75 (73–78)	72 (65–78)	81 (78–84)
Household crowding, % (95% CI) above median people (>2) per room^a^	57 (55–60)	69 (64–74)	NA
Household poverty score, % (95% CI) above median score^b^	50 (47–53)	63 (57–68)	NA
Anthropometry			
Overweight, % (95% CI) above median BMI (>25 kg/m^2^)^c^	48 (45–50)	46 (40–52)	12 (10–14)

Abbreviations: BMI, body mass index; CI, confidence interval; NA, not applicable (because these variables were assessed at the household rather than individual level).

^a^ A continuous measure of crowding was calculated by people sleeping in the house divided by number of rooms in the house. The median of this continuous crowding variable was exactly 2 people per room. The variable “household crowding” refers to the percentage of household's containing more people per room than the cohort median (2). When splitting into above and below this median, “2” cannot be split and therefore those houses with exactly 2 people per room were apportioned to the “crowded” (ie, above the median) households. This results in 57% of cohort households being above the cohort median people per room and thus crowded.

^b^ The variable household poverty score refers to the percentage of households with a poverty score above the household median.

^c^ The variable “overweight” refers to the percentage of individuals whose BMI was above the median BMI of the entire cohort of tuberculosis-exposed cohabitants and patients, and is the same as that defined by the World Health Organization (>25 kg/m^2^).

**Table 2.** Seasonality of Tuberculosis Risk Factors, Tuberculosis Infection, and Tuberculosis Disease

	Peak Season and Dates			6-mo Proportions		
	Season Start Date	Peak Date	Season End Date	Peak Season	Rest of Year	*P* Value
Tuberculosis risk factors						
Household crowding, % (n/N) [95% CI]	8 Apr	8 Jul (midwinter)	7 Oct	58.9% (206/350) [53.7–64.0]	46.2% (171/370) [41.1–51.3]	<.001
Hours without direct sunlight, % (n/N) [95% CI]	20 May	18 Aug (midwinter)	18 Nov	92.7% (15 622/16 848) [92.3–93.2]	74.4% (11 746/15 792) [73.7–75.1]	<.001
Vitamin D deficient (<50 nmol/L), % (n/N) [95% CI]	24 May	23 Aug (midwinter)	22 Nov	66.7% (58/87) [56.8–76.6]	49.1% (53/108) [39.6–58.5]	.01
Tuberculosis infection						
TST positivity, % (n/N) [95% CI]	8 Jul	7 Oct (late winter)	6 Jan	62.5% (388/621) [58.7–66.3]	54.7% (420/768) [51.2–58.2]	.003
IGRA positivity, % (n/N) [95% CI]	4 Oct	3 Jan (early summer)	4 Apr	59.1% (166/281) [53.3–64.8]	50.5% (149/295) [44.8–56.2]	<.04
Tuberculosis disease						
Tuberculosis symptom onset incidence, % (n/N) [95% CI]^a^	1 Dec	2 Mar (midsummer)	1 Jun	0.14% (416/307 623) [.12–.15]	0.12% (361/307 623) [.11–.13]	<.05
Tuberculosis diagnosis incidence, % (n/N) [95% CI]^b^	24 Dec	24 Mar (late summer)	23 Jun	0.15% (466/307 623) [.14–.17]	0.13% (390/307 623) [.11–.14]	<.01

The peak seasons for the tuberculosis risk factors, tuberculosis infection, and tuberculosis disease and the proportion occurring during the peak season versus the rest of the year is shown.^c^

Abbreviation: CI, confidence interval.

^a^ Tuberculosis symptom onset was calculated using longest duration of symptoms, including cough (with or without phlegm or blood), weight loss, fever, and night sweats. More general symptoms (eg, headache and nausea) were not included in symptom-onset calculations. Incidence is shown as the total number of people with onset of symptoms that were subsequently diagnosed to be caused by laboratory-proven pulmonary tuberculosis over four 6-month periods as a percentage of the total population of the study site estimated in a national census during the study period.

^b^ Incidence is shown as the total number of people diagnosed with laboratory-proven pulmonary tuberculosis over four 6-month periods as a percentage of the total population of the study site estimated in a national census during the study period.

**Figure 2.**
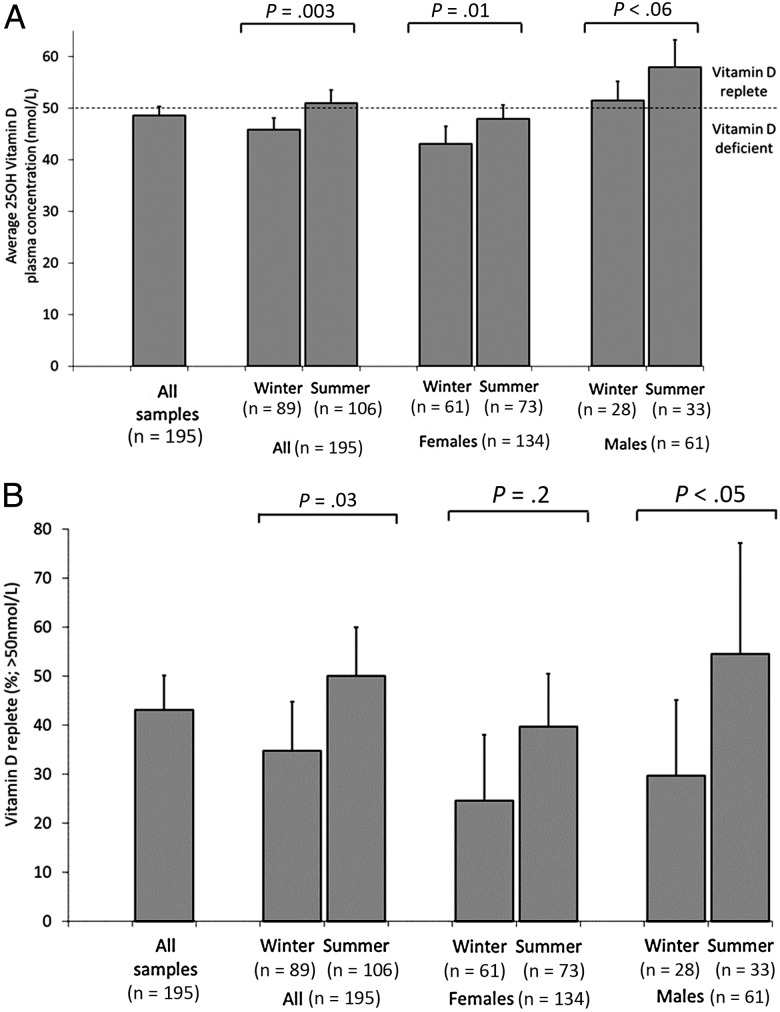
Vitamin D status for the entire study population (n = 195) by sex and season. *A*, Vitamin D plasma concentration. *B*, Vitamin D concentration replete (ie, 25OHD concentrations ≥50 nmol/L). Bars represent 95% confidence intervals. Abbreviation: 25OHD, calcifediol.

^c^ These observed 6 monthly actual count data differ slightly from the 6-month moving average data shown in Figure 2 because of differences in the way these data are calculated.

For all participants, height and weight were measured and body mass index (BMI) was calculated. Socioeconomic position was measured using a composite household poverty index incorporating 13 variables, including education, housing, services, and assets [27]. Crowding was measured by number of people per room, calculated as the number of people sleeping in the house divided by the total number of rooms in the house.

At enrollment, all tuberculosis-exposed cohabitants were asked to undergo testing for latent tuberculosis with TST as described [33]. From 7 March 2005 to 9 November 2006, additional resources became available that allowed all consecutive recruited tuberculosis-exposed cohabitants who agreed to provide a blood sample to also undergo IGRA using the QuantiFERON Tuberculosis-Gold assay (Cellestis). From this date, 68% (n = 576) of cohabitants agreed to provide a blood sample and had interpretable IGRA results. As recommended by the manufacturer, IGRA results were reported qualitatively as positive, negative, or indeterminate, not quantitatively.

### Vitamin D Concentrations

Of the tuberculosis-exposed cohabitants enrolled in the micronutrient supplementation trial who had TST and IGRA results, a subset (n = 102) selected using random-number tables provided blood samples at recruitment. These tuberculosis-exposed cohabitants stated that they were not taking micronutrient supplements. Those tuberculosis-exposed cohabitants who did not receive micronutrient supplements also provided blood samples 1 (n = 48) and 6 months (n = 45) postrecruitment. Therefore, a total of 195 (102 + 48 + 45) blood samples were taken. Vitamin D concentrations were measured by radio-immunoassay using inductively coupled mass spectrometry. Plasma concentrations of <50 nmol/L vitamin D were considered to be deficient and ≥50 nmol/L replete [22].

### Statistical Analysis

Raw data and 6-month moving averages (a series of averages of time-series data subsets used to highlight longer-term trends/cycles) were analyzed to examine seasonal variation and divided into “peak” season (3 months prior to and after the peak value for that variable) and “the rest of the year” (the remaining 6 months). Power calculations for sample size were not performed. Continuous data with a Gaussian distribution were summarized as means with their 95% confidence intervals (CIs) and compared by Student *t* test. Continuous data with a non-Gaussian distribution were converted to categorical data above or below the median value (for crowding 2 people/room and for poverty arbitrary units were used), summarized as proportions with their 95% CIs and compared with the *Z* test of proportions. For multiple regression analyses, noncontributory variables were removed in a backwards-stepwise manner according to the likelihood-ratio test. Relative risks (RRs) were calculated using generalized linear model and binomial analysis. The regression model was repeated using season as both a categorical variable (summer versus winter) and as a continuous variable (days from trough hours of direct sunlight). Tuberculosis incidence was calculated using the population of Ventanilla of 307 623 people estimated by national census during the study period. All *P* values were 2-sided, and analyses, including calculation of population-attributable fractions (PAFs), used the Stata program (version 12).

## RESULTS

### Study Population

The study population is summarized in Table 1. Among participants, tuberculosis-exposed cohabitants were older, had lower education level, were more often female, and were more likely to be overweight than tuberculosis patients (all *P* < .05). There was a median household average of 2.0 (interquartile range [IQR], 1.5–3.0) people per room.

### Vitamin D

Figure 2*A* shows the average vitamin D concentrations and Figure 2*B* the proportion of vitamin D replete samples, both analyzed by season and sex. The mean vitamin D concentration was 48.6 nmol/L (95% CI, 46.9–50.3).

The proportion of samples that were vitamin D replete (≥50 nmol/L) was 43.1% (95% CI, 36.1–50.1). During summer, the average vitamin D concentration was 51.0 nmol/L and significantly higher than during the winter (45.8 nmol/L; *P* < .003, Figure 2*A*). Male sex and summer were significantly associated with greater likelihood of being vitamin D replete (Figure 2*B*).

The randomly selected subgroup of tuberculosis-exposed cohabitants who had vitamin D assays were more likely to be poor (*P* = .0006) and have more crowding (*P* < .003) than the entire cohort of tuberculosis-exposed cohabitants. However, neither poverty nor crowding were associated with vitamin D concentrations or being vitamin D replete (all *P* > .1, Table 3). There were no other differences between this subgroup and the other tuberculosis-exposed cohabitants.

**Table 3.** Regression Analysis of Associations With Vitamin D Levels

	Vitamin D 25OHD Plasma Concentrations (nmol/L), Linear Regression				Vitamin D Replete (≥50 nmol/L), Binomial Regression				
	Univariate Regression		Multiple Regression		Univariate Regression		Multiple Regression		
	Coefficient (95% CI)	*P* Value	Coefficient (95% CI)	*P* Value	Relative Risk (95% CI)	*P* Value	Adjusted PAF	Relative Risk (95% CI)	*P* Value
Sex (male)	9.3 (4.4–14)	<.001	9.3 (5.8–13)	<.001	2.00 (1.4–2.9)	<.001	24 (9–36)	2.0 (1.4–2.8)	<.001
Season (summer)^a^	5.2 (2.2–8.3)	.001	5.3 (2.0–8.5)	<.001	1.4 (1.0–2.0)	.03	19 (1–33)	1.4 (1.0–1.9)	<.05
Age; years	0.11 (−.088–.30)	.2	…	…	1.0 (.99–1.0)	.3	…	…	…
Any postprimary education	2.0 (−2.8–6.8)	.4	…	…	1.3 (.83–2.2)	.2	…	…	…
Household crowding, above median people per room	−1.2 (−6.2–3.9)	.6	…	…	1.0 (.68–1.6)	.9	…	…	…
Household poverty score, above median score	−1.006 (−5.7–3.7)	.6	…	…	0.78 (.53–1.2)	.2	…	…	…
Overweight, above median BMI (>25 kg/m^2^)^b^	3.1 (−1.9–8.1)	.2	…	…	1.1 (.73–1.6)	.7	…	…	…

This table shows the results of linear regression of vitamin D plasma concentrations in nmol/L as the outcome variable and binomial regression with odds of being vitamin D replete (≥50 nmol/L) as the outcome variable.

All analyses presented above were clustered by individual because some individuals had more than 1 blood sample taken. Specifically, 102 tuberculosis-exposed cohabitants provided blood samples at recruitment, and 48 and 45 of these individuals provided blood samples again at 1 and 6 months postrecruitment, respectively. Therefore, a total of 195 blood samples were taken. Blank cells indicate variables that did not meet the criteria for inclusion in the multiple regression analysis.

Abbreviations: 25OHD, calcifediol; adjusted PAF, population-attributable fraction derived from multiple logistic regression using the “aflogit” function of STATA; BMI, body mass index; CI, confidence interval.

^a^ In addition to the analysis of season shown, when univariate and multiple linear regression analyses were repeated using “days from trough in hours of direct sunlight” as a continuous variable in place of “season,” male sex and days from trough in hours of direct sunlight remained associated with greater likelihood of being vitamin D replete or having higher vitamin D concentrations.

^b^ BMI indicates weight in kilograms divided by height in meters squared.

### Seasonal Association of Crowding, Sunlight, Vitamin D Deficiency, and Tuberculosis

**Figure 3.**
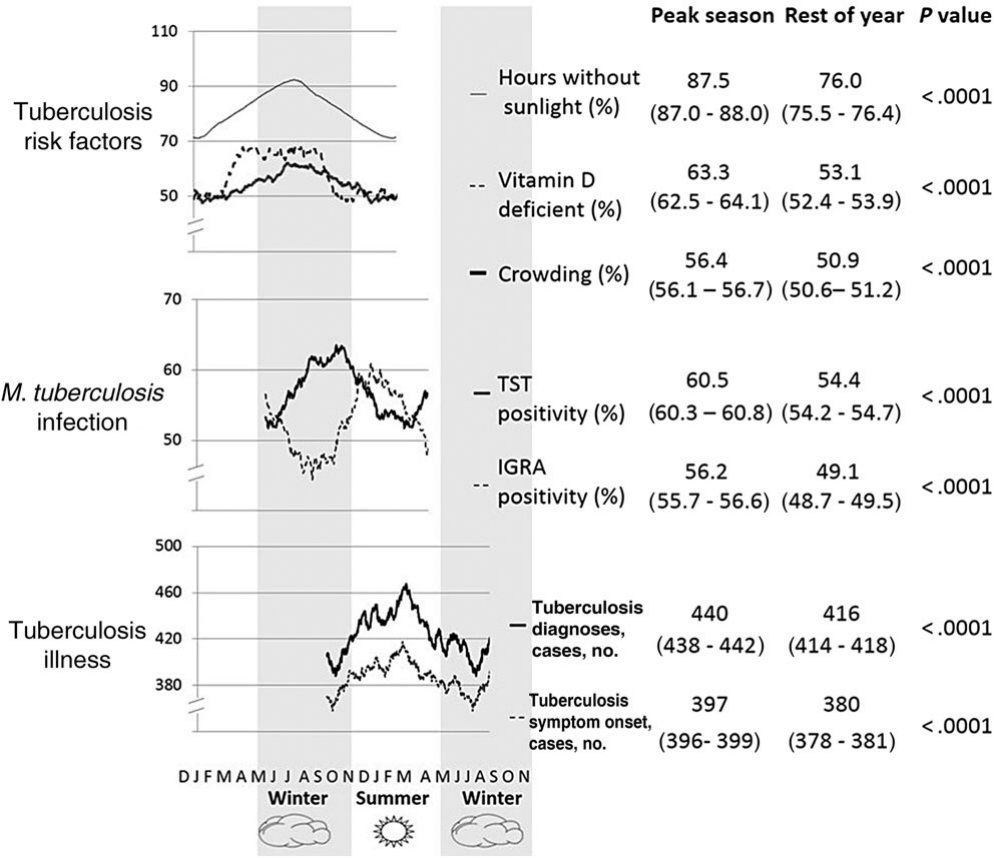
Schematic demonstrating the seasonality of tuberculosis risk factors in midwinter, infection in late winter and early summer, and disease in midsummer. Letters represent months of the year. Trend lines represent 6-month moving averages of raw data that differ slightly from the 6 monthly actual counts shown in Table 2, owing to the latter being raw data. In the “tuberculosis risk factors” section, hours without sunlight is represented by the thin continuous black trend line, vitamin D deficiency by the dashed black line, and crowding by the thick continuous black trend line. The numbers stated for the incidence of tuberculosis symptom onset and tuberculosis diagnoses are the 6-month moving average data corresponding to those in Table 2. Abbreviations: IGRA, interferon-γ release assay; TST, tuberculin skin test.

Figure 3 demonstrates the seasonality of tuberculosis risk factors, infection, and illness: the midwinter peak in putative tuberculosis risk factors (crowding, hours without direct sunlight, and vitamin D deficiency), the sequential late-winter (TST) and early-summer (IGRA) peak in tuberculosis infection, and finally the midsummer peak in tuberculosis symptom onset followed after 3 weeks by subsequent tuberculosis diagnosis. Table 2 shows the analysis by season of crowding, hours without direct sunlight, vitamin D deficiency, tuberculosis infection, and illness.

### Tuberculosis Risk Factors

#### Crowding

The peak in proportion of households with crowding occurred in midwinter in July prior to both the peak in hours without direct sunlight and vitamin D deficiency (Figure 3). During the 6 months with most crowding, the proportion of crowded households was 13% points higher than the rest of the year (*P* < .001, Table 2).

#### Hours Without Direct Sunlight

The peak in proportion of hours without direct sunlight occurred in August, midwinter, as can be seen in Figure 3. The proportion of hours without direct sunlight was 19% points higher in winter than summer (*P* < .001, Table 2).

#### Vitamin D Deficiency

Vitamin D deficiency was detected in 56.9% (111/195) of samples (Table 2). Figure 3 demonstrates that the peak proportion of samples with vitamin D deficiency occurred in midwinter in the week following the peak in hours without direct sunlight. During the 6 months around this peak, the proportion of samples that were vitamin D deficient was 17% points higher than the rest of the year (*P* = .01, Table 2).

### Tuberculosis Infection in Tuberculosis-Exposed Cohabitants

#### TST

Six weeks following the peak in vitamin D deficiency, the peak in the proportion of positive TST results occurred in late winter (Figure 3). During the 6 months around this peak, the proportion of positive TST results was 8% points higher than the rest of the year (*P* = .003, Table 2).

#### IGRA

The peak in the proportion of positive IGRA tests occurred in early summer, 12 weeks following the peak in proportion of positive TST results. During the 6 months around the IGRA peak, the proportion of positive IGRA tests was 9% points higher than the rest of the year (*P* < .04, Table 2).

### Tuberculosis Disease in Laboratory-Proven Tuberculosis Patients

#### Tuberculosis Symptom Onset

Five months after the peak in tuberculosis infections as indicated by TST, the peak in tuberculosis symptom onset occurred in midsummer (Figure 3). During the 6 months around this date, 14% more patients had tuberculosis symptom onset than the rest of the year (*P* < .05, Table 2).

#### Tuberculosis Diagnosis

The peak in tuberculosis diagnosis occurred in late summer, 3 weeks following the peak in tuberculosis symptom onset (Figure 3). During the 6 months around this date, 13% more patients were diagnosed with tuberculosis than the rest of the year (*P* < .01, Table 2).

### Regression Analyses

Table 3 shows the multiple regression analysis of the association between vitamin D concentrations (and the likelihood of being vitamin D replete) and the characteristics of the study population. Male sex (RR 2.0, PAF 24%; *P* < .001) and summer (RR 1.4, PAF 19%; *P* < .05) were associated with greater likelihood of being vitamin D replete (ie, lower likelihood of deficiency). To assess the robustness of these findings, additional analyses of vitamin D concentrations (instead of being vitamin D replete) were performed and showed the same pattern of significance (Table 3; Figure 2).

## DISCUSSION

Vitamin D deficiency was common in this high-risk group of tuberculosis-exposed people, more common in females, and peaked in midwinter, shortly after peak crowding and hours without direct sunlight. This was followed by a peak in tuberculosis infections in late winter and, after the known 5-month median tuberculosis incubation period [34], by a peak in tuberculosis symptoms in midsummer. Finally, after the 3-week interval required for tuberculosis case finding in this setting [35], tuberculosis diagnoses subsequently peaked in late summer. These findings suggest that seasonal vitamin D deficiency and crowding may explain the previously enigmatic interval from the midwinter peak in tuberculosis risk factors until the late-summer peak in tuberculosis diagnoses.

Vitamin D deficiency was found in over half of this Peruvian cohort of tuberculosis-exposed cohabitants. Deficiency associated closely with hours without direct sunlight as reported in temperate climates in Europe [36] and North America [37]. Defining vitamin D deficiency is controversial [20]. We selected the threshold of vitamin D concentrations <50 nmol/L, as recently suggested in international guidance and relevant studies [22, 38]. While such a threshold may be suitable at a population level to predict diseases like osteomalacia, it may fail to detect significant linear associations between vitamin D concentrations and nonskeletal disease risk (such as type-2 diabetes, ischemic heart disease, or cancer) [39]. Therefore, we examined vitamin D as both a categorical *and* linear-dependent variable, and found the results to be concordant.

Female sex was associated with greater likelihood of vitamin D deficiency, independent of season. This is important because international vitamin D supplementation guidelines do not generally differentiate between gender, apart from pregnancy or lactation. The predominance of vitamin D deficiency in females in our study may relate to genetic predisposition or diet. However, the most likely explanation may be difference in behavior because in this setting, men spend more time outside working [40].

Vitamin D deficiency is a biologically plausible risk factor for tuberculosis infection and disease because it suppresses immune responses specific to tuberculosis infection [17] and has been epidemiologically associated with tuberculosis disease [11, 24]. The active metabolite of vitamin D (1,25-dihydroxyvitamin D) upregulates the cellular vitamin D receptor to inhibit mycobacterial growth [41] and increases cathelicidin expression by macrophages, which promotes mycobacterial cell death [15–17]. Therefore, increasing vitamin D concentrations in spring may potentially lead to a “seasonal immune reconstitution” with increased granuloma formation, tissue inflammation, and corresponding symptoms in people subsequently diagnosed with tuberculosis in summer. Moreover, although current in vivo evidence suggests no effect [22, 23], there is some in vitro evidence that vitamin D supplements may improve the treatment response of patients with tuberculosis disease [24]. Work in a similar Peruvian population has demonstrated that vitamin D receptor polymorphisms were associated with tuberculosis patients' time to sputum culture conversion [42]. Thus, vitamin D deficiency, immunology, and genetic factors imply a role in tuberculosis susceptibility.

The temporal association we observed between peak crowding and vitamin D deficiency followed by tuberculosis infection as indicated by the peak in TST positivity extends previous TST conversion findings in tuberculosis-exposed cohabitants in Spain [18]. The 12-week interval we observed from peak TST positivity until the peak IGRA positivity may be explained by differences in time to conversion between these tests: the optimum timing of IGRA testing remains to be defined [43] and in tuberculosis outbreaks, IGRA conversion occurred 3 [44] to 6 months [45] after exposure, often later than TST conversion [46]. Moreover, serial IGRA analysis has shown high rates of both initial conversion and subsequent reversion in healthcare workers without known tuberculosis exposure, complicating interpretation [47]. However, our study did not measure TST or IGRA conversion but the proportion of positive tests at different cross-sectional time points. A final possible explanation for the difference between IGRA and TST is that their accuracy for tuberculosis infection may be influenced by vitamin D concentrations: work in the study setting has shown that other micronutrients affect TST sensitivity [48]. Thus, the temporal discrepancy we found between seasonal proportion of positive IGRA and TST tests is novel and the mechanisms behind such a discrepancy require further exploratory research.

The seasonality of winter vitamin D deficiency and summer tuberculosis diagnoses that we characterized in tuberculosis-exposed cohabitants extends findings in tuberculosis patients in Europe [19] and South Africa [11]. Low vitamin D concentrations and more vitamin D deficiency in midwinter may have led to increased host-susceptibility to tuberculosis infection seen in late winter and to the subsequent progression to tuberculosis disease, after a 5-month incubation period. This 5-month incubation period is the same as that found in a study that used DNA fingerprinting to accurately identify progression from household tuberculosis exposure to tuberculosis infection and disease [34]. Although our findings are relevant to both, we were unable to determine which episodes of tuberculosis disease were a result of recent tuberculosis infection versus reactivation of latent tuberculosis. However, other recent studies showed that tuberculosis seasonal variation was more pronounced in children and clustered cases, suggesting recent infection as the more likely explanation [14].

The interaction of social determinants along the causal pathway from tuberculosis exposure to infection and disease is complex and likely relates to increased transmission (greater exposure through crowding, increased time spent indoors, and poorer ventilation [29]), susceptibility (poorer nutrition, lower immunity), and marginalization (health-seeking behavior, education). Our study measured crowding, potential tuberculosis exposure that in winter may contribute to the seasonality we observed. Crowding is a known marker of poverty and both crowding and poverty are independently associated with tuberculosis [29, 30]. However, crowding is complex, poorly defined, and specific to geographical settings [31]. Research in high-resource countries has defined crowding as >1 person/room and severe crowding as >1.5 people/room [32], which would have classified virtually all of our households as crowded, preventing meaningful analysis. The World Health Organization suggests that measuring floor space [31] can be problematic and that it may be more appropriate to consider crowding as above the midpoint number of persons/room. We used this a priori definition as a locally appropriate crowding definition in this current research and also in ventilation research that is being published separately. Our finding of more crowding in winter than summer is novel. The reasons behind this crowding seasonality require further investigation and may include economic migration (seasonal employment), schooling, public holidays, and selling food produce after harvests. As noted in another recent study [49], it is unlikely that crowding and increased transmission in winter alone is the factor responsible for tuberculosis seasonality. Our findings suggest that both crowding and vitamin D deficiency are independently associated with tuberculosis seasonality.

Temporal associations cannot prove causation, and other confounding factors may have contributed to tuberculosis seasonality. For example, diet was not examined in our study. However, there is little variation in foodstuff availability and consumption in Ventanilla, and BMI was used as a proxy nutrition indicator. Climate-related confounding factors could include temperature [13], humidity, rain, and climate change. Despite high humidity year round, annual rainfall in Lima is very low (10–30 mm) and there is minimal variation in temperature (average 14°C in winter and 20°C in summer). An important confounder not measured in the present study was seasonal variation in healthcare seeking and access. Healthcare-seeking behavior may vary with work-market, harvest, or school season, as may provision of medical care at health posts and hospitals. Such variations may extend to the number of people tested for tuberculosis. Other confounding factors include: concomitant respiratory-tract infections, smoke inhalation, and air quality. With regard to vitamin D concentrations, the inductively coupled mass spectrometry assay we used is now recognized to be prone to interlaboratory variations, and isotope-dilution liquid chromatography tandem mass spectrometry may be preferable [50] but was not widely available during our study. In addition to this ecological analysis, it would be valuable to perform larger future studies to test for associations between baseline vitamin D concentrations in tuberculosis-exposed household contacts and subsequent progression to tuberculosis disease, although such a study would be confounded by the seasonality of vitamin D concentrations that we report here. Vitamin D concentrations were measured in a random subset of tuberculosis-exposed cohabitants who happened to be poorer and live in more crowded households than all tuberculosis-exposed cohabitants. This chance occurrence does not appear to have been important because multiple regression demonstrated that neither crowding nor poverty were independently associated with vitamin D concentrations or being vitamin D replete.

In conclusion, the sequential peaks in midwinter crowding, vitamin D deficiency, tuberculosis infection, tuberculosis symptom onset, and finally late-summer tuberculosis diagnoses potentially explain the previously enigmatic seasonality of tuberculosis. These findings suggest that climate change and recommendations to reduce the risk of skin cancer by avoiding sun exposure may influence tuberculosis susceptibility. The associations that we have identified between season, crowding, vitamin D, and tuberculosis emphasize the potential for correcting vitamin D deficiency and mitigating poverty to contribute to tuberculosis prevention.
